# Impacts of Unhealthy Behaviors on Mental Health among Public Health Residents: The PHRASI Study

**DOI:** 10.3390/diseases12070134

**Published:** 2024-06-27

**Authors:** Vincenza Gianfredi, Lorenzo Stacchini, Giuseppa Minutolo, Valentina De Nicolò, Nausicaa Berselli, Angela Ancona, Alessandro Catalini, Fabrizio Cedrone

**Affiliations:** 1 Department of Biomedical Sciences for Health, University of Milan, via Pascal, 36, 20133 Milan, Italy; vincenza.gianfredi@unimi.it; 2 Department of Health Science, University of Florence, 50121 Firenze, Italy; 3 Food Hygiene, Nutritional Surveillance and Prevention, Department of Prevention, Provincial Healthcare Authority of Palermo, 90129 Palermo, Italy; giuseppa.minutolo@asppalermo.org; 4 Department of Public Health and Infectious Diseases, Sapienza University of Rome, 00161 Rome, Italy; valentina.denicolo@uniroma1.it; 5 Department of Biomedical, Metabolic and Neural Sciences, University of Modena and Reggio Emilia, Via Campi, 287, 41125 Modena, Italy; nausicaa.berselli@unimore.it; 6 School of Public Health, Vita-Salute San Raffaele University, 20132 Milan, Italy; ancona.angela@hsr.it; 7 UOC Igiene degli Alimenti e Nutrizione, Dipartimento di Prevenzione, AST Macerata, 62100 Macerata, Italy; alecata@icloud.com; 8 Hospital Management, Local Health Authority of Pescara, 65100 Pescara, Italy

**Keywords:** medical residents, depression, anxiety, alcohol abuse, physical activity, sleep satisfaction, unhealthy behaviors

## Abstract

Unhealthy behaviors (UBs) can affect mental health. The most impactful of these are alcohol abuse, sedentary behavior, and sleep disturbances. Since these UBs are not well explored in medical residents, this study aims to overcome this gap, focusing on outcomes such as depression and anxiety. The Public Health Residents’ Anonymous Survey in Italy (PHRASI) is a nationwide cross-sectional study based on an 88-item questionnaire. UBs were explored through validated questionnaires: the Alcohol Use Disorders Identification Test-c (AUDIT-C), the International Physical Activity Questionnaire (IPAQ), and the Insomnia Severity Index (ISI). Depressive symptoms and anxiety were detected by Patient Health Questionnaire-9 (PHQ-9) and Generalized Anxiety Disorder-7 (GAD-7), respectively. Hierarchical cluster analysis described clusters using responses from the AUDIT-C, IPAQ, and ISI. A total of 375 participants were included in this study. Three distinct clusters (CL) were identified: CL1 was characterized by a moderate risk for alcohol abuse and high insomnia dissatisfaction, with a significantly higher frequency of depressive symptoms (46%, *p* < 0.001) and anxiety (53%, *p* < 0.001); CL2 had no risk for alcohol abuse, high sleep satisfaction, and better mental health profile, with the lowest prevalence of depressive symptoms (15%, *p* < 0.001); CL3 had the highest risk of alcohol abuse, the highest rate of physical activity (40%, *p* = 0.013), and the lowest prevalence of anxiety (27%, *p* < 0.001). Subjects belonging to CL1 required much more attention to prevent the worsening of mental outcomes. Interventions for promoting mental health should be addressed in all Public Health schools to create more sustainable working conditions.

## 1. Introduction

Certain habits and lifestyles, such as being sedentary and alcohol abuse, can have a negative impact on health with relevant outcomes [1,2]. Although a standardized and unique definition does not yet exist [1,2,3], these are considered unhealthy behaviors (UBs), including any behavior that can represent a threat to health and psychophysical integrity [3]. Subjects can have one predominantly unhealthy behavior (UB) or more than one simultaneously, which can have a dramatic and variable impact on mental health [1,2].

The global prevalence of alcohol abuse, a leading UB, is estimated to affect millions of individuals, with the World Health Organization (WHO) reporting that harmful alcohol use results in 3 million deaths annually [4]. Among medical professionals, the prevalence of alcohol use has been variably reported. Some studies have reported that medical residents may consume alcohol at higher rates than other healthcare professionals, a worrying trend given the stressful nature of medical training and its potential impact on mental health [5].

Physical inactivity, another critical UB, contributes significantly to the global incidence of non-communicable diseases, including mental health disorders [6].

Epidemiological data suggest that insufficient physical activity is prevalent in 23% of the global population, a figure that mirrors trends observed among healthcare professionals, including those in training [7]. The sedentary demands of medical education and clinical duties further exacerbate this issue, highlighting the need for interventions aimed at increasing physical activity among medical residents.

Sleep disturbances, encompassing issues such as insomnia, poor sleep quality, and short sleep duration, have been linked to an increased risk of mental health problems. Epidemiological studies have shown a high prevalence of sleep disorders among healthcare workers, with significantly higher rates of insomnia and poor sleep quality among medical residents compared to the general population [8]. This relationship is of particular concern given the rigorous demands of medical training and the critical role of sleep in cognitive function and overall well-being.

Mental health issues among medical professionals, especially depression and anxiety, have emerged as significant occupational hazards, with prevalence rates exceeding those in the general population [9]. Recent studies indicate that up to one-third of medical residents report experiencing symptoms of depression or depressive disorders—a rate approximately two to five times higher than that observed in the general population [10]. Anxiety disorders are also highly prevalent, with studies suggesting rates of 20–30% among medical residents compared with 18.1% in the general adult population [11].

Public Health residents (PHRs), tasked with the dual responsibilities of clinical training and public health advocacy, may face unique stressors that influence their engagement in UBs and impact their mental health. Unlike residents in other medical specializations, PHRs must balance population health, preventive measures, and healthcare policies, adding an additional layer of complexity to their training, potentially affecting their lifestyle choices and mental health. In particular, the dual responsibilities of clinical training and public health advocacy can lead to unique stressors such as the need to balance rigorous academic requirements with fieldwork in diverse and often resource-limited settings. Moreover, PHRs might be more susceptible to physical inactivity compared to other residents due to the nature of their work, which often involves extensive time spent on research, policy development, and public health initiatives. These activities can be more sedentary compared to the clinical duties of other medical residents, who may have more physically demanding roles. Given these unique stressors, PHRs may be particularly vulnerable to engaging in unhealthy behaviors such as alcohol use, physical inactivity, and experiencing sleep disturbances. However, despite a growing body of evidence on the detrimental effects of unhealthy behaviors and their impact on mental health, significant gaps remain in understanding these issues specifically among Public Health residents. Previous research has predominantly focused on the general population or broadly across medical professionals, often failing to account for the unique stressors experienced by PHRs. Moreover, the existing literature often fails to account for the multifaceted nature of UBs and their complex interactions with mental health outcomes. Many studies have explored the impact of individual UBs, such as alcohol consumption or physical inactivity, in isolation, neglecting the cumulative and interactive effects of multiple UBs on mental health. This oversight underscores the need for a more holistic approach to understanding how combinations of UBs impact the well-being of medical residents. By adopting a holistic approach, we seek to provide new insights into the cumulative impact of multiple unhealthy behaviors on mental health, highlighting the unique vulnerabilities of PHRs.

This study aims to address gaps in the existing literature by providing epidemiological insights into the prevalence of UBs among PHRs in Italy and exploring their association with mental health outcomes. While previous studies, such as those by Suvarna et al. (2020) and Zhang et al. (2022) [12,13], have examined the impact of adverse behaviors on mental health, our study distinguishes itself by focusing specifically on a group of medical professionals in training who play a crucial role in public health promotion. These data suggest that health professionals might engage in unhealthy behavior on a global level; therefore, by situating our study within this international context, we can draw comparisons and highlight the unique stressors faced by PHRs in Italy, thereby making our findings more relevant to readers from other regions.

By shedding light on these relationships within this specific group of medical professionals, the study seeks to contribute to the development of targeted strategies for promoting healthier lifestyles and improving mental health among future public health leaders. Furthermore, the findings can influence public health policies and practices by providing evidence for specific interventions aimed at supporting the well-being of Public Health residents, thereby enhancing the overall effectiveness of public health services.

## 2. Materials and Methods

### 2.1. Study Design and Participants Recruitment

The Public Health Residents’ Anonymous Survey in Italy (PHRASI) is a nationwide cross-sectional study aimed at examining various aspects of mental health among Italian Public Health medical residents [14]. This study was voluntary and self-administered via an electronic survey created using Google Forms (©2022 Google, Mountain View, CA, USA), targeting approximately 1600 residents enrolled in four-year courses at Italian postgraduate Public Health schools. Methodological details have been previously described. The minimum required sample size, calculated using Charan and Biswas’s formula for cross-sectional studies, was determined to be 315 participants [15]. To prevent missing data, all 88 questionnaire items were set as mandatory in Google Forms.

The survey link was disseminated through the mailing list of the medical residents’ Assembly of the Italian Society of Hygiene and Preventive Medicine. Additionally, representatives from each postgraduate Public Health school were contacted to further distribute the survey among their peers.

Data collection took place from 14 June 2022, to 26 July 2022. All collected data were stored in a password-protected, anonymous computerized database accessible only to the researchers. Because the survey was anonymous and respondents could not be identified, ethical committee approval was not required. The responses were analyzed in aggregate form, adhering to Italian and European personal data management laws [16,17,18].

### 2.2. Variables of Interest

Physical activity has been assessed using the Italian short version of the International Physical Activity Questionnaire (IPAQ) [19,20]. The IPAQ is a validated tool that includes seven questions designed for adults aged 15–69 years. It inquiries about the type (vigorous, moderate, and walking) and frequency (days per week and time per day) of physical activity over the past seven days. The questionnaire provides separate scores for walking, moderate-intensity activity, and vigorous-intensity activity, calculated as a continuous variable. Then, physical activity can be categorized according to the Guidelines for Data Processing and Analysis of the IPAQ [21]. In the cluster identification process, the three scores for each level of intensity activity were used as continuous variables, while physical activity in the outcomes was expressed as a categorical variable. Physical activity level was categorized as highly active if it met any one of the following two criteria: vigorous-intensity activity on at least 3 days and accumulating at least 1500 metabolic equivalent task (MET)-minutes/week; or 7 or more days of any combination of walking, moderate-intensity or vigorous intensity activities achieving a minimum of at least 3000 MET-minutes/week. Minimally active was defined as any one of the following three criteria: 3 or more days of vigorous activity for at least 20 min per day; 5 or more days of moderate-intensity activity or walking for at least 30 min per day; or 5 or more days of any combination of walking, moderate-intensity or vigorous intensity activities, achieving a minimum for at least 600 MET-min/week. Inactive was defined as whoever had the lowest level of physical activity and did not meet the criteria for the above-mentioned categories.

Alcohol abuse was assessed using the Italian translation of the Alcohol Use Disorders Identification Test-C (AUDIT-C) [22]. The AUDIT-C is a validated, three-item short version of the AUDIT questionnaire [23]. Each item is scored on a scale of 0 to 4. A final score of five or higher for males, and four or higher for females, indicates a potential risk for alcohol abuse. 

Sleep satisfaction was measured using the second item of the Insomnia Severity Index (ISI2), “How satisfied are you with your current sleep pattern?” [24,25]. The result was expressed on a five-point Likert-type scale ranging from 0 (very satisfied) to 4 (very dissatisfied).

### 2.3. Mental Health Outcomes

Depressive symptoms were assessed using the Italian version of the Patient Health Questionnaire-9 (PHQ-9) [26]. The PHQ-9 score was calculated according to standard guidelines. This questionnaire consists of nine items, each rated on a four-point scale from 0 (“not at all”) to 3 (“nearly every day”). The scores for each item are summed to produce a continuous total score ranging from 0 (no symptoms) to 27 (all symptoms present nearly every day). A PHQ-9 score of 10 or above indicates clinically relevant (moderate to severe) depressive symptoms. Conversely, a score below 10 is rarely seen in individuals with major depression and suggests no, minimal, or mild depressive symptoms.

Anxiety was assessed using the Italian translation of the Generalized Anxiety Disorder-7 (GAD-7) questionnaire [27]. GAD-7 is a validated screening tool that evaluates the severity of symptoms associated with the four most common anxiety disorders: generalized anxiety disorder, panic disorder, social phobia, and post-traumatic stress disorder. The questionnaire consists of seven items asking participants how often they have experienced various anxiety-related problems over the past two weeks. An overall score of 3 or higher suggests the possible presence of an anxiety-related disorder.

### 2.4. Covariates

The sociodemographic characteristics analyzed included age, sex, region of residence, cohabitation status, number of children, region of traineeship, course year in the postgraduate program, off-site status, commuter status, willingness to work at the current workplace or training site after completing the postgraduate course, simultaneous attendance at two traineeships, intention to retake the entrance test for a different postgraduate program or general practitioner course, having a compatible employment contract with the postgraduate program, and the ability to make ends meet with their income.

Age was treated as a continuous variable. The number of children was categorized as “0” for no children, “1” for one child, and “>1” for more than one child. The regions of residence and traineeship were classified into “North”, “Center”, and “South and Islands” according to the definitions of the Italian National Institute of Statistics (ISTAT). Specifically, the “North” included Aosta Valley, Liguria, Lombardy, Piedmont, Emilia-Romagna, Friuli-Venezia Giulia, Trentino-Alto Adige/Südtirol, and Veneto; the “Center” included Lazio, Marche, Tuscany, and Umbria; and the “South and Islands” included Abruzzo, Apulia, Basilicata, Calabria, Campania, Molise, Sardinia, and Sicily.

The remaining variables were dichotomized as follows: cohabitation was categorized into “living alone” or “living with others” (including roommates or family members); course years in the postgraduate program were divided into the first and second biennium; willingness to continue working at the current site after the postgraduate course was classified as “yes” for those who responded “absolutely yes” or “more yes than no” and “no” for the opposite responses; having a compatible employment contract with the postgraduate program was categorized as “yes” or “no”, irrespective of the specific contract type; and the ability to make ends meet with their income was dichotomized as “easily” for those who responded “easily” and “hardly” for those who indicated “with someone’s help” or “with difficulties”.

### 2.5. Statistical Analysis

Sociodemographic and behavioral variables were described using descriptive statistics, presenting means and standard deviations (SD) for continuous variables, whereas frequencies and percentages were used for categorical variables. Responses to IPAQ, AUDIT-c, and ISI2 were used to identify clusters with distinct UBs profiles through a hierarchical cluster analysis. No variable showed missing values. To identify the best number of clusters, Ward’s clustering criterion based on Manhattan distance [28] was used to calculate the dissimilarity matrix. A Hubert index was graphed to choose the optimal number of clusters. Supplementary Figure S1 provides a graph of that index, the corresponding knee, and related statistics. Additional indices were consulted using the R package NbClust, identifying three as the optimal number of clusters based on thirty algorithms. The clusters were shown in a dendrogram. To ensure the stability of the clustering results, we conducted multiple iterations of the clustering process and observed consistent formation of three clusters. We also performed silhouette analysis, which measures how similar an object is to its own cluster compared to other clusters. The average silhouette width supported the choice of three clusters, indicating a clear and stable separation between clusters. We examined the practical relevance and interpretability of the identified clusters by analyzing the characteristics of the data within each cluster. The composition of the clusters was described using a Kruskal–Wallis test for continuous variables or a Chi-square test for categorical variables; each were performed to evaluate whether significant differences in the distribution of UBs and all the collected covariates existed between clusters. Statistical significance was set at *p* < 0.05. All the analyses were performed using R 4.2.3.

## 3. Results

### 3.1. Participant Demographics

**Figure 1:**
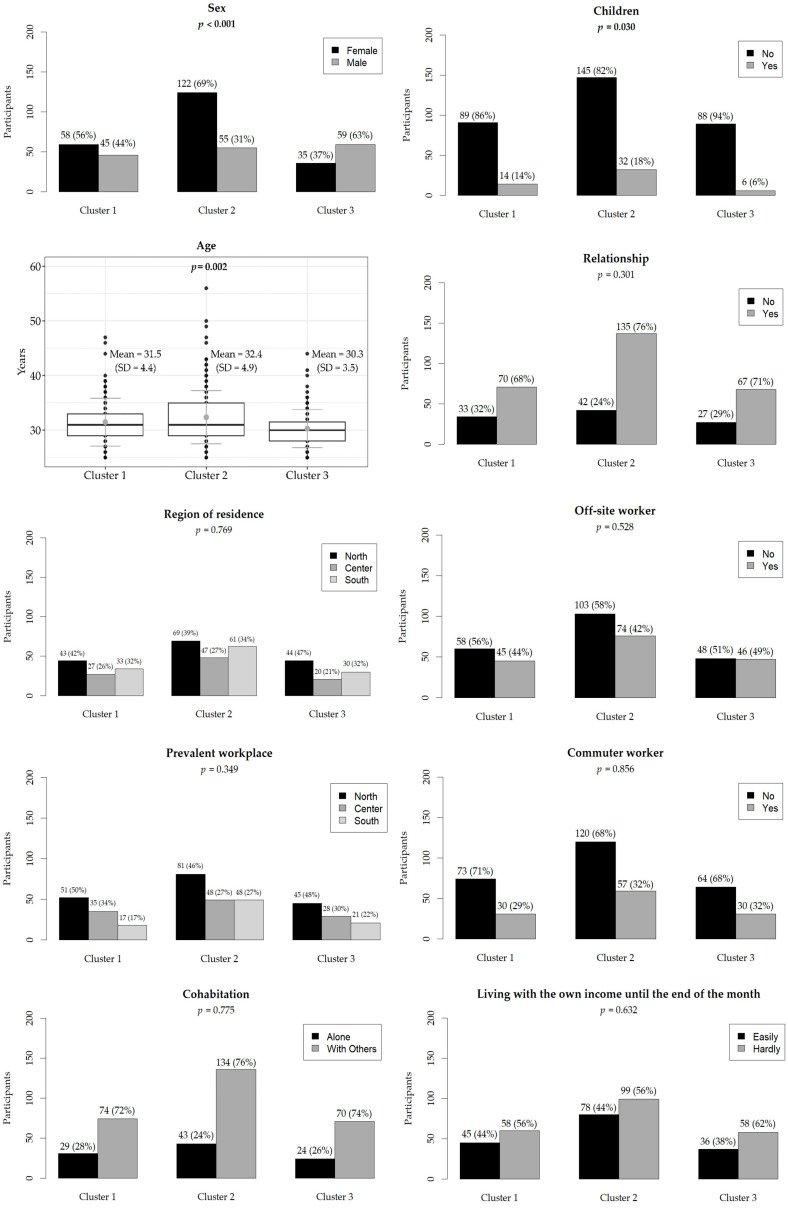
Demographic characteristics of participants. The relative frequencies are in brackets. The boxplot shows the mean (light gray dot) and standard deviation (dark gray line) of age in years. Statistically significant *p*-values are in bold.

**Figure 2:**
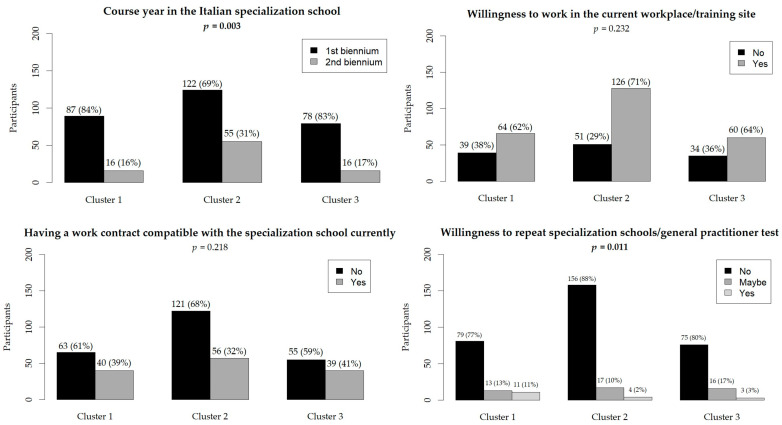
Participants’ working conditions. The relative frequencies are in brackets. Statistically significant *p*-values are in bold.

**Figure 3:**
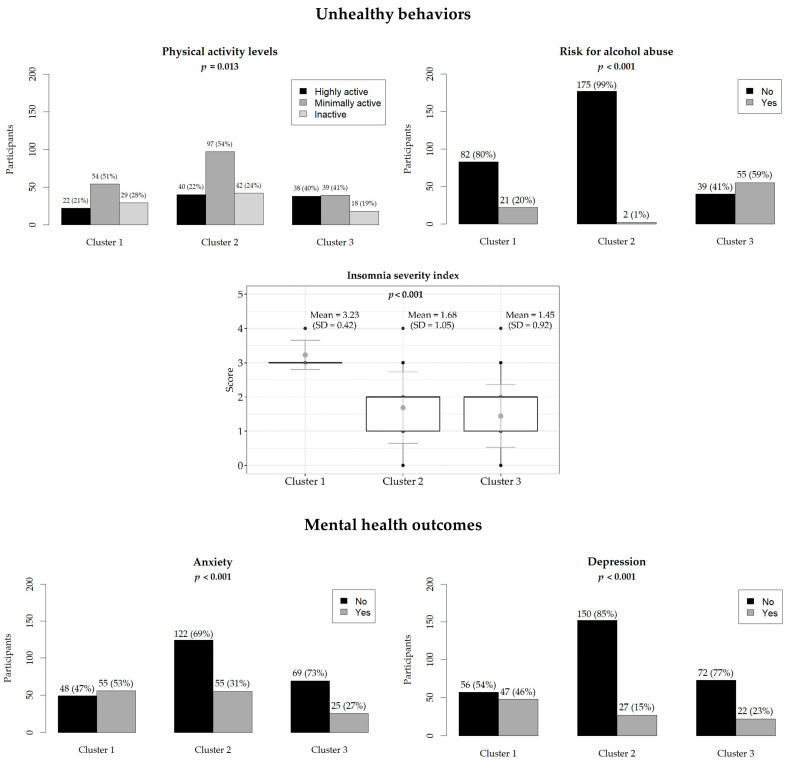
Participants’ unhealthy behaviors and mental health outcomes. The relative frequencies are in brackets. The boxplot shows the mean (light gray dot) and standard deviation (dark gray line) of the ISI score. Statistically significant *p*-values are in bold.

A total of 375 Public Health residents participated in the PHRASI study, providing a diverse representation across various regions of Italy. The demographic breakdown revealed a balanced gender distribution, with 57% female participants. The median age of respondents was 31 years. The hierarchical cluster analysis, based on reported behaviors regarding alcohol consumption, physical activity, and sleep satisfaction, identified three distinct clusters (CL) among the participants: CL1 (N = 103), CL2 (N = 177), and CL3 (N = 94). Results are shown in Figure 1, Figure 2 and Figure 3.

The region of residence did not show significant differences (*p* = 0.769), with a relatively even distribution across the center, north, and south in all clusters. Similarly, prevalent workplace locations did not differ significantly (*p* = 0.349). Living conditions, including cohabitation status (*p* = 0.775) and the number of children (*p* = 0.053), were not significantly different among clusters. Most participants in all clusters cohabited with others, and a majority had no children. There were no significant differences in the proportion of off-site workers (*p* = 0.528) and commuter workers (*p* = 0.856) among the clusters. Additionally, the ease of living on one’s income until the end of the month was similar across clusters (*p* = 0.632), with approximately half of the participants in each cluster reporting difficulty. Having additional compatible contracts with the specialization school did not differ significantly among clusters (*p* = 0.218). Willingness to work at their current workplace or training site (*p* = 0.232) also showed no significant differences, although there was a trend towards higher willingness in CL2 (71%). Results are shown in Figure 1, Figure 2 and Figure 3.

### 3.2. Hierarchical Cluster Analysis Outcomes 

**Figure 4:**
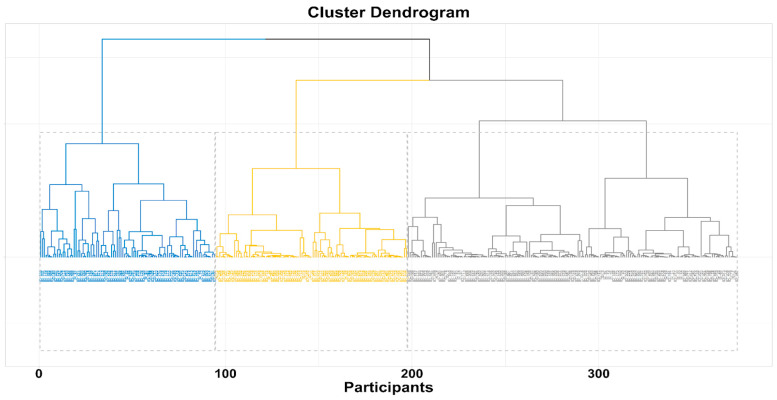
Hierarchical cluster dendrogram-based onward.

CL1 (moderate risk for alcohol abuse and high insomnia severity) comprised 103 residents (56% female, median age 31); this cluster was characterized by a significant proportion living with others (72%) and mostly having no children (86%). They faced moderate challenges living on their income (56%) and had notable mental health concerns, including screening positive for depression (16% with clinically relevant depressive symptoms) and anxiety (53%). Moreover, they had higher insomnia severity (ISI2 = 3), moderate prevalence of alcohol abuse (20%), and were minimally active (51%). They were primarily in the early stages of their specialization training (84% at the first biennial of residency), with a significant number not having additional contracts (61%) (Figure 4).

CL2 (no risk for alcohol abuse, high sleep satisfaction, and better mental health profile) comprised 177 participants, predominantly female (69%), with a median age of 31 years. They mostly resided and worked in the North (39%) and lived with others. A significant majority did not have children (82%). Economic conditions were challenging for many, with a notable proportion finding it difficult to live on their income (56%). Most were in the early stages of their specialization training (69%) and many of them did not have additional contracts (68%). Mental health indicators showed the lowest prevalence of clinically relevant depressive symptoms (15%) of all the other clusters and a lower frequency of anxiety (31%) than CL1. Further, very few of them were at risk for alcohol use disorder (1%) and were satisfied with their sleep quality; however, 54% were minimally physically active. 

CL 3 (highest risk of alcohol abuse) encompassed 94 residents, predominantly male (63%), with a median age of 30 years. They primarily resided (47%) and worked (48%) in the North and lived with others (74%). A significant majority had no children (94%). Economic conditions were challenging for many, with a higher proportion finding it difficult to live on their income (62%). Most were in the early stages of their specialization training (83%), with a notable percentage having additional contracts (41%). Mental health indicators showed a moderate prevalence of depression (23%) and the lowest prevalence of anxiety (27%) of all the other clusters. Additionally, a substantial proportion were at risk for alcohol use disorders (59%). Physical activity levels were relatively high with 40% of subjects being highly physically active, and insomnia severity was generally low among this group.

In summary, significant differences were found between the clusters in terms of sex distribution, age, mental health indicators, and certain aspects of educational and occupational conditions, highlighting distinct demographic and psychological profiles within each cluster.

## 4. Discussion

The PHRASI study successfully gathered data from 375 Public Health residents across Italy, achieving a balanced representation in terms of region and gender. The median age of participants was 31 years. Hierarchical cluster analysis, based on behaviors related to alcohol consumption, physical activity, and sleep satisfaction, identified three distinct clusters among the participants: CL1 (N = 103), CL2 (N = 177), and CL3 (N = 94). This analysis provides insight into the demographic and behavioral characteristics of each cluster.

### 4.1. Interpretation of Findings

The findings of the PHRASI study, which reveal a significant association between UBs and mental health outcomes among Public Health residents, resonate with a growing body of research on the mental health challenges faced by medical professionals. The identification of specific clusters based on UBs, including alcohol abuse, physical activity and sleep satisfaction, and their differential impact on mental health, offers a nuanced addition to the literature, which has traditionally focused on broader medical populations without distinction by speciality or residency type. In detail, our results highlight significant associations between all the UBs considered—alcohol abuse, unsatisfactory sleep, and physical inactivity—and mental health outcomes among Public Health residents in Italy. The identification of three distinct clusters based on UBs provides a nuanced understanding of the risks and protective factors influencing the mental health of this specific population. Clusters at higher risk of alcohol abuse (CL1 and CL3) exhibited notably higher levels of depression and anxiety, aligning with existing evidence that links substance abuse with poorer mental health outcomes. Moreover, it should be considered that substance use (including alcohol), is prevalent among young adults [29], particularly among high-pressure groups, as healthcare workers [30]. Conversely, clusters characterized by higher sleep satisfaction (CL2 and CL3) and a high level of physical activity demonstrated lower levels of these mental health issues, emphasizing the protective role of adequate rest and physical activity. However, it is crucial to consider potential confounding variables that might affect these relationships, such as workload, social support, and access to mental health resources. For instance, PHRs with heavier workloads and less social support may be more prone to unhealthy coping mechanisms like alcohol abuse, which in turn exacerbates mental health issues. Conversely, those with stronger social support networks might engage more in protective behaviors like physical activity and adequate sleep, which buffer against stress and promote better mental health outcomes.

The association between alcohol use and increased risk of depression and anxiety identified in this study is consistent with previous research conducted among various medical specialities. For instance, a systematic review by Oreskovich et al. found that physicians in the United States reported higher rates of alcohol abuse or dependence compared to the general population [31]. However, the PHRASI study extends this knowledge by specifically highlighting the prevalence of alcohol abuse among PHRs in Italy, suggesting that the stress and demands unique to this speciality may contribute to higher alcohol consumption as a coping mechanism. Furthermore, variations in workload and social support among residents may also play a significant role in these observed patterns.

Similarly, the protective role of satisfactory sleep against depression and anxiety observed in our study aligns with findings from Tsuno et al. [32], which emphasized the importance of sleep quality among healthcare professionals [33]. The PHRASI study contributes to this area by providing evidence from the Italian Public Health residency context, underscoring the need for targeted sleep hygiene interventions within this group. It is also plausible that workload and shift patterns significantly impact sleep quality, suggesting that interventions should also address these structural factors.

Moreover, while the relationship between physical activity and mental health is well documented in the general population, research focusing on medical residents, such as that by Weight et al. [34], has indicated a significant gap between recommended physical activity levels and actual practices. Our study’s finding of significant differences in physical activity levels between clusters confirms the beneficial role of physical activity on mental health outcomes [35,36]. It is plausible to think that medical residents’ engagement in physical activity is influenced by several factors, including work schedules, stress levels, and availability of resources, highlighting the need for targeted interventions to bridge this gap [37]. Additionally, the role of institutional support in providing opportunities for physical activity should be considered.

The nuanced findings of the PHRASI study also complement comparative studies, like that of Mata et al. [38], which found that medical residents experienced a higher prevalence of depressive symptoms compared to the general population. By focusing on PHRs, the current study not only corroborates the elevated risk for mental health issues within the medical profession but also emphasizes the variability of that risk across different residency programs and unhealthy behaviors. Additionally, variations in workload and social support among different residency programs may further explain these differences.

Lastly, our findings contribute to the limited literature on UBs and mental health among Public Health professionals in international contexts. Studies from other countries, such as the work of Shanafelt et al. in the United States, have explored burnout and mental health across medical specialities, but few have specifically addressed Public Health residents [39]. The PHRASI study fills this gap by providing insights from Italy, highlighting both the universal and unique challenges faced by PHRs in different healthcare systems.

### 4.2. Potential Biological Mechanisms

The observed relationships between UBs and mental health outcomes can be partially explained by underlying biological mechanisms. For instance, alcohol abuse is known to affect neurotransmitter systems in the brain, particularly those involved in the regulation of mood, such as serotonin and dopamine [40]. This disruption can lead to symptoms of depression and anxiety, as these chemicals play a crucial role in mood regulation and emotional stability [40]. Similarly, inadequate sleep can impair the body’s stress response system, including the hypothalamic–pituitary–adrenal (HPA) axis, leading to increased stress sensitivity and the exacerbation of mental health symptoms [41,42]. This means that when sleep is insufficient, the body’s ability to handle stress diminishes, which can negatively impact mental health [43]. Dysregulation of the HPA axis can also be driven by physical inactivity [44]. Regular physical activity helps regulate the HPA axis, and a lack of exercise can lead to its dysfunction, further increasing stress sensitivity and worsening mental health outcomes [45]. Moreover, physical inactivity, along alcohol misuse, and poor sleep have been associated with systemic inflammation, which has been implicated in the pathophysiology of depression and anxiety [46,47]. These data suggest that the above-mentioned unhealthy behaviors contribute to mental health issues through inflammatory processes. In addition to these negative impacts, regular exercise offers protective benefits for mental health. It enhances neuroplasticity by increasing brain-derived neurotrophic factor (BDNF) levels, which supports the growth and maintenance of neurons. Exercise also supports balanced autonomic nervous system function, which is essential for maintaining a stable mood and reducing anxiety. Furthermore, regular physical activity improves sleep quality, maintains metabolic health, and reduces oxidative stress, all of which are beneficial for mental well-being [48,49,50]. 

These biological pathways highlight the complex interplay between lifestyle behaviors and mental health. They suggest that interventions aimed at improving UBs could have a beneficial impact on the mental well-being of Public Health residents. Addressing factors such as alcohol misuse, physical inactivity, and poor sleep through targeted interventions could mitigate their adverse effects on mental health, leading to improved overall health and quality of life for this population.

### 4.3. Implications for Public Health Policies

The PHRASI study’s findings highlight the need for Public Health residency programs to foster environments that proactively support mental health. Educational institutions and healthcare organizations should collaborate to develop and implement comprehensive wellness programs that are specifically tailored to the needs of PHRs. To implement these recommendations, several specific plans can be outlined. First, mandatory workshops and training sessions focusing on stress management and coping strategies for dealing with the unique pressures of Public Health work [51]. These workshops would include evidence-based techniques and interactive sessions to help residents recognize and manage stress effectively. Additionally, by integrating training in mindfulness and resilience-building techniques into the residency curriculum, institutions can demonstrate a commitment to the well-being of their residents, potentially mitigating the impact of UBs on mental health [52].

Given the significant association between alcohol abuse, physical inactivity, sleep dissatisfaction, and mental health outcomes identified in the study, public health policies should aim to address these behaviors directly [53]. Policies could include the implementation of screening programs within residency institutions to identify individuals at risk for alcohol abuse and sleep disorders early in their training. Using validated tools for regular screenings, followed by immediate referrals to appropriate services, can ensure timely intervention [54]. Early intervention strategies, such as counselling, peer support groups, and access to mental health services, can be integral components of a comprehensive approach to wellness. Furthermore, advocating for changes in work schedules to ensure adequate rest and recovery time can help improve sleep hygiene among residents, contributing to better overall mental health. Work schedule reforms are essential to ensure residents have adequate rest and recovery time. Policies should be implemented to limit the number of consecutive working hours and mandate rest periods between shifts. This can help improve sleep hygiene among residents, contributing to better overall mental health.

At the same time, promoting physical activity is a critical strategy for improving mental health. Public Health policies should encourage the creation of physical activity programs that are accessible and appealing to residents, such as group fitness classes, walking meetings, and incentives for active commuting [55]. Additionally, policy efforts to integrate physical activity breaks into the workday can help mitigate the sedentary nature of much of the residency work, providing both mental and physical health benefits.

The findings underscore the importance of advocating for increased resources dedicated to mental health support within the medical community, particularly for those in Public Health specialities. This could involve lobbying for the allocation of funding towards mental health services, including the hiring of more counsellors and psychologists who specialize in working with healthcare professionals. This can be facilitated through policy directives and the inclusion of mental health goals in institutional strategic plans. Engaging stakeholders, including residency program directors, mental health professionals, and residents, in the design and implementation of wellness programs is essential. Policies aimed at reducing the stigma associated with seeking mental health care can also encourage residents to take advantage of available resources without fear of judgment or professional repercussions [56].

Lastly, the study’s implications extend beyond immediate interventions to suggest long-term strategies for incorporating mental health considerations into the core of Public Health education and practice. Developing policies that integrate mental health promotion into public health campaigns, community health initiatives, and preventive medicine can foster a culture of wellness that benefits not only Public Health residents but also the communities they serve. This holistic approach recognizes that the health of Public Health professionals is intrinsically linked to their ability to effect positive change in public health outcomes. The expected effects include improved mental health outcomes, reduced incidence of unhealthy behaviors, and a more resilient and effective Public Health workforce. These changes will not only benefit the residents but also positively impact the communities they serve by fostering a culture of wellness and holistic care.

### 4.4. Future Research Directions

The cross-sectional design of the PHRASI study provides valuable insights but cannot establish causality between UBs and mental health outcomes. Future research should employ longitudinal study designs to track changes in UBs and mental health over time among PHRs. Such studies could provide a more nuanced understanding of the temporal relationships between these factors, identify critical periods of vulnerability, and assess the long-term impact of interventions aimed at mitigating UBs. From this perspective, interventional studies testing the effectiveness of specific strategies designed to reduce UBs and improve mental health outcomes are extremely needed. Moreover, while the PHRASI study focuses on Public Health residents, future research should explore how UBs and mental health outcomes vary across different medical specialities. Moreover, while the study provides valuable insights for Italian Public Health residents, the findings may not be directly generalizable to other populations or regions without further study. Considerations of cultural, systemic, and educational differences would be necessary for broader application of the results. Future research should examine these factors to determine how the findings can be adapted and applied in different contexts, ensuring that interventions are culturally and regionally appropriate. Comparative studies could help identify whether the challenges observed among Public Health residents are unique or shared with residents in other fields and in other countries. Understanding these differences can inform speciality-specific interventions and support systems.

### 4.5. Limitations and Strengths

Before generalizing our results, several limitations should be acknowledged. The data may be subject to social desirability and recall biases due to the self-reported nature of the measures, despite assurances of anonymity [57]. However, previous research has shown that online, anonymous surveys are less prone to social desirability bias when compared to paper-based studies [58]. Additionally, residents who were more isolated or dissatisfied with their postgraduate course may have been under-represented, potentially underestimating the prevalence of depressive symptoms and their association with job dissatisfaction. Although PHQ-9 and GAD are validated screening tools for depressive symptoms and anxiety, they cannot make a diagnosis, although their high sensitivity and specificity mitigate the risk of misclassification. The cross-sectional design also limits the ability to establish causality or temporality, restricting the analysis to prevalence estimates. Moreover, another potential limitation of our study is the absence of certain anthropometric data, such as physical health measures and weight. The inclusion of these data could have provided a more comprehensive analysis of how physical health interacts with psychological health, sources of stress, and other key variables. This limitation may affect the robustness of our findings and the ability to fully understand the correlations between variables. However, despite this limitation, a key strength of our study is the comprehensive collection of detailed psychosocial and behavioral data, which provide valuable insights into the mental health risks and factors affecting public health residents. Future research should aim to include a broader range of physical health data to enhance the overall analysis and provide a more holistic understanding of the factors influencing mental health. Lastly, one notable limitation of our study is the absence of comprehensive personality assessments. While we utilized validated screening tools capable of identifying individuals at higher risk of certain mental health symptoms, we did not assess personality traits or diagnose mental disorders. The inclusion of these detailed personality assessments would have provided a more holistic view of the participants’ personality structure and functioning, both functional and dysfunctional. As a result, our data may present a biased view, as it does not cross-reference individual personality characteristics with the mental health symptoms detected. Future research should incorporate comprehensive personality assessments to enhance the robustness of the findings and confirm the results obtained. Despite this limitation, our study offers valuable preliminary data on mental health risks, serving as an essential first step in identifying individuals who may benefit from further, more detailed evaluations and interventions.

Despite these limitations, the study has notable strengths. It is the first to investigate depressive symptoms among PHRs in Italy, making it unique and original. The study achieved high nationwide participation with an even distribution across Italy’s regions, and the sample size exceeded the minimum requirement [59]. The survey was tailored specifically for PHRs and disseminated through targeted channels, ensuring accurate data collection with no missing data. Validated instruments like PHQ-9 enhanced the reliability of findings, and statistical rigor was ensured through hierarchical cluster analysis [60].

## 5. Conclusions

The PHRASI study represents a significant step forward in understanding the prevalence of UBs and their association with mental health outcomes among PHRs in Italy. Using a cross-sectional design and hierarchical cluster analysis, the study identified three distinct clusters of residents differentiated by their engagement in UBs. These clusters were differentiated based on their engagement in UBs such as alcohol use, levels of physical activity, and sleep quality, as well as their corresponding levels of depression and anxiety. While the study’s findings highlight the importance of addressing these behaviors and their underlying factors to potentially improve the mental well-being of Public Health professionals, it is important to acknowledge the limitations of our research. The cross-sectional nature of the study does not allow for causal inferences, and the absence of detailed personality assessments may limit the depth of our conclusions. Despite these limitations, the study provides a critical foundation for developing targeted interventions aimed at improving the mental health and overall well-being of Public Health professionals, ultimately enhancing their capacity to effectively contribute to the public health mission. By addressing these behaviors and their underlying factors, there is potential to significantly improve the mental well-being of Public Health professionals, ultimately enhancing their ability to contribute effectively to the Public Health mission.

## Data Availability

Authors can be contacted for information about the data presented.

## Supplementary Materials

The following supporting information can be downloaded at: https://www.mdpi.com/article/10.3390/diseases12070134/s1, Supplementary Figure S1. The Hubert index plotted to determine the optimal number of clusters. The graph includes the index values, the identified knee point, and related statistical data.
