# Supplementary material for: Impacts of Unhealthy Behaviors on Mental Health among Public Health Residents: The PHRASI Study
Source: Diseases. 2024 Jun 27;12(7):134. doi: 10.3390/diseases12070134 (PMC11275475; doi:10.3390/diseases12070134)

Supplementary Figure S1. The Hubert index plotted to determine the optimal number of clusters. The graph includes the index values, the identified knee point, and related statistical data.

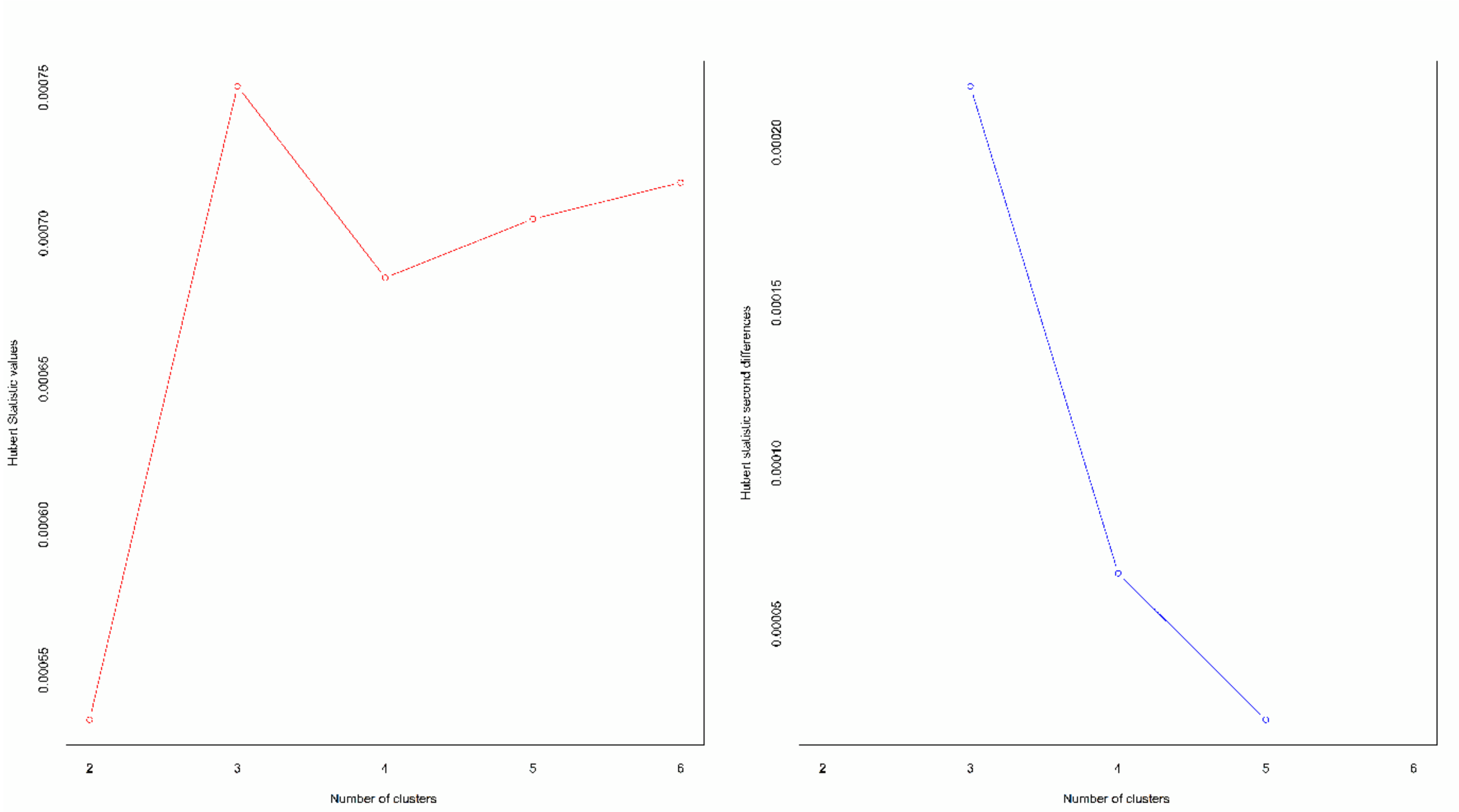

Supplement: Supplementary file 1 [file diseases-12-00134-s001.zip › diseases-3037604-supplementary.pdf]
